# Assembly and mother centriole recruitment of IFT-B subcomplexes to form IFT-B holocomplex

**DOI:** 10.1247/csf.25027

**Published:** 2025-06-24

**Authors:** Koshi Tasaki, Yohei Katoh, Hye-Won Shin, Kazuhisa Nakayama

**Affiliations:** 1 Department of Physiological Chemistry, Graduate School of Pharmaceutical Sciences, Kyoto University, Sakyo-ku, Kyoto 606-8501, Japan

**Keywords:** cilia, ciliogenesis, distal appendages, IFT-B complex, mother centriole

## Abstract

For the biogenesis and maintenance of cilia, bidirectional protein trafficking within cilia is crucial, and is conducted by intraflagellar transport (IFT) trains containing the IFT-A and IFT-B complexes that are powered by dynein-2 and kinesin-II motors. We have recently shown that before the assembly of anterograde IFT trains, the IFT-A, IFT-B, and dynein-2 complexes are independently recruited to the mother centriole/basal body. The IFT-B complex, which consists of 16 subunits, can be divided into the IFT-B1 and IFT-B2 subcomplexes, and IFT-B1 can be further divided into the IFT-B1a and IFT-B1b subgroups. Here we investigated how the IFT-B complex is assembled and recruited to the mother centriole for ciliogenesis. Analyses using cells with knockouts of individual IFT-B subunits, and analyses of proteins coimmunoprecipitated with EGFP-fused IFT-B2, IFT-B1b, and IFT-B1a subunits expressed in these knockout cells demonstrated the following: (i) although IFT-B2 is dispensable for the linkage between IFT-B1b and IFT-B1a, it is essential for their localization to the mother centriole; (ii) IFT-B1b is essential both for bridging IFT-B2 and IFT-B1a, and for their localization to the mother centriole; (iii) IFT-B1a is not required for the linkage between IFT-B2 and IFT-B1b nor for their localization to the mother centriole; and (iv) all IFT-B components (IFT-B2, IFT-B1b, and IFT-B1a) are essential for ciliogenesis. Thus, although ciliogenesis is not a prerequisite for the recruitment of the IFT-B complex to the mother centriole, the linkage between IFT-B2 and IFT-B1b is crucial for the mother centriole localization of the IFT-B complex for ciliogenesis.

## Graphical Abstract

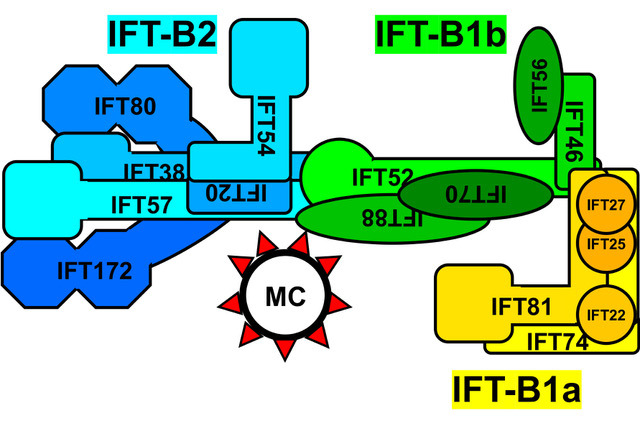

## Introduction

Primary cilia are antenna-like organelles that sense and transduce extracellular signals, such as the Hedgehog (Hh) family of morphogens, and play crucial roles in embryonic development and adult organ homeostasis (Anvarian *et al.*, 2019; Hilgendorf *et al.*, 2024; Kopinke *et al.*, 2021). Their structure is based on axonemal microtubules that extend from the basal body derived from the mother centriole, which has distal appendages (DAPs) with nine-fold symmetry, and are surrounded by the ciliary membrane (Breslow and Holland, 2019; Kumar and Reiter, 2021). To perform their sensory functions, specific signaling proteins are present in the cilioplasm and on the ciliary membrane, such as G protein-coupled receptors, including Smoothened and GPR161, which regulate Hh signaling positively and negatively, respectively (Anvarian *et al.*, 2019; Kopinke *et al.*, 2021; Schou *et al.*, 2015). The distinct composition of ciliary proteins is achieved by the transition zone (TZ) at the ciliary base, which functions as a barrier controlling the import and export of soluble and membrane proteins (Garcia-Gonzalo and Reiter, 2017; Park and Leroux, 2022).

The biogenesis of cilia and their maintenance rely on the intraflagellar transport (IFT) machinery, which was first discovered in *Chlamydomonas* flagella, and is often referred to as IFT trains or IFT particles (Cole *et al.*, 1998; Kozminski *et al.*, 1993, 1995; Piperno and Mead, 1997). The IFT machinery consists of two main multisubunit complexes (Nakayama and Katoh, 2020; Taschner and Lorentzen, 2016). The IFT-A complex is composed of six subunits, and participates in retrograde protein trafficking within cilia driven by the dynein-2 complex, and in the import of membrane proteins across the TZ together with the TULP3 adaptor protein. The IFT-B complex is composed of 16 subunits, and participates in anterograde trafficking driven by heterotrimeric kinesin-II, and in the export of membrane proteins across the TZ along with the BBSome complex, which is composed of eight Bardet-Biedl syndrome (BBS) proteins.

Recent studies of *Chlamydomonas* flagella using cryoelectron microscopy and cryoelectron tomography revealed that the anterograde IFT trains are composed of repetitive units of the IFT-B complex and the IFT-A complex that are located between the IFT-B repeats and the ciliary membrane; the IFT-B/IFT-A stoichiometry is approximately 2:1. The dynein-2 complex is then loaded onto the assembled trains as an anterograde IFT cargo (Jordan *et al.*, 2018; Lacey *et al.*, 2023; Toropova *et al.*, 2019; van den Hoek *et al.*, 2022).

The 16 subunits of the IFT-B complex can be grouped into two subcomplexes; namely, the IFT-B1 subcomplex (IFT22/IFT25/IFT27/IFT46/IFT52/IFT56/IFT70/IFT74/IFT81/IFT88) and the IFT-B2 subcomplex (IFT20/IFT38/IFT54/IFT57/IFT80/IFT172) (see Fig. 5A) (Boldt *et al.*, 2016; Katoh *et al.*, 2016; Lucker *et al.*, 2005; Petriman *et al.*, 2022; Richey and Qin, 2012; Taschner *et al.*, 2011, 2016). The two subcomplexes are connected by a tetramer unit involving IFT52 and IFT88 from IFT-B1, and IFT38 and IFT57 from IFT-B2; this tetramer also constitutes the binding site for heterotrimeric kinesin-II (Funabashi *et al.*, 2018). The IFT-B1 subcomplex can be further divided into two subgroups, i.e., IFT-B1a (IFT22/IFT25/IFT27/IFT74/IFT81) and IFT-B1b (IFT46/IFT52/IFT56/IFT70/IFT88), which are connected via an interaction between the IFT74–IFT81 dimer from IFT-B1a and the IFT46–IFT52 dimer from IFT-B1b (Katoh *et al.*, 2016; Petriman *et al.*, 2022; Taschner *et al.*, 2014; Zhou *et al.*, 2022). The IFT-B complex is connected to IFT-A by an interaction involving IFT122–IFT144 from IFT-A and IFT52–IFT88 from IFT-B (Hesketh *et al.*, 2022; Ishida *et al.*, 2021; Kobayashi *et al.*, 2021; Lacey *et al.*, 2023). Among the IFT-B subunits, IFT74 and IFT81 form a tight heterodimer via their long coiled-coil regions, and have been shown to play a crucial role in transporting tubulin dimers to support the extension and maintenance of axonemal microtubules (Bhogaraju *et al.*, 2013; Kubo *et al.*, 2016). Therefore, depletion or knockout (KO) of any of the IFT-B subunits, including IFT74 and IFT81, often results in a cell phenotype that lacks cilia (Nakayama and Katoh, 2018).

**Fig. 1:**
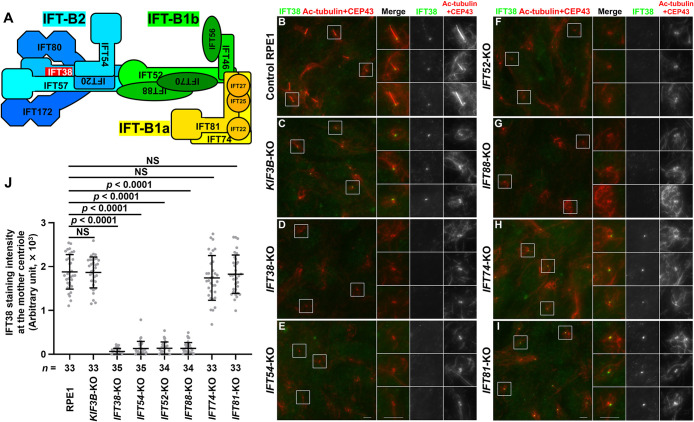
Components of IFT-B2 and IFT-B1b, but not IFT-B1a, are required for mother centriole recruitment of IFT38 (A) Model diagram of assembly of the IFT-B complex with IFT38 highlighted in red. The beginning of the subunit name indicates the N-terminal side. (B–I) Control RPE1 (B), *KIF3B*-KO (C), *IFT38*-KO (D), *IFT54*-KO (E), *IFT52*-KO (F), *IFT88*-KO (G), *IFT74*-KO (H), and *IFT81*-KO (I) cells were cultured under serum-starved conditions for 24 h to induce ciliogenesis, and immunostained with antibodies against IFT38 and acetylated α-tubulin (Ac-tubulin) + CEP43 (CEP43 was previously referred to as FOP/FGFR1OP). Images shown on the right side are 2.5-times enlarged images of the boxed regions. Scale bars, 5 μm. (J) Staining intensity of IFT38 at the mother centriole of various cells was measured and shown as scatter plots. Dots indicate individual samples, and horizontal lines and error bars indicate means and SDs, respectively. Total numbers of analyzed cells from a single experiment are shown (*n*). The immunostaining experiment was repeated three times to confirm the reproducibility. Statistical significances among multiple groups were calculated using one-way ANOVA followed by Tukey’s multiple comparison test. NS, not significant.

**Fig. 2:**
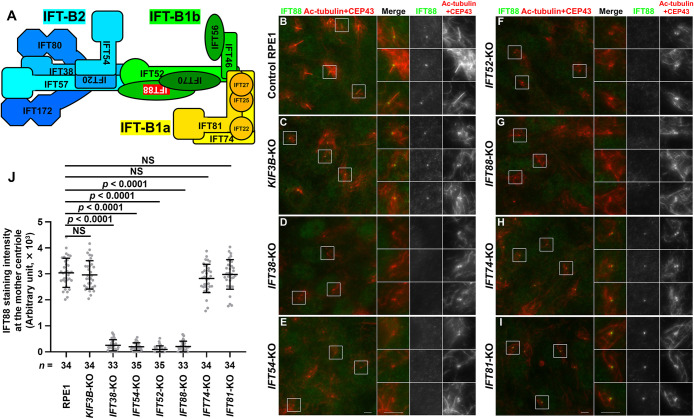
Components of IFT-B2 and IFT-B1b, but not IFT-B1a, are required for mother centriole recruitment of IFT88 (A) Model diagram of assembly of the IFT-B complex with IFT88 highlighted in red. (B–I) Control RPE1 (B), *KIF3B*-KO (C), *IFT38*-KO (D), *IFT54*-KO (E), *IFT52*-KO (F), *IFT88*-KO (G), *IFT74*-KO (H), and *IFT81*-KO (I) cells were cultured under serum-starved conditions for 24 h, and immunostained with antibodies against IFT88 and Ac-tubulin + CEP43. Images shown on the right side are 2.5-times enlarged images of the boxed regions. Scale bars, 5 μm. (J) Staining intensity of IFT88 at the mother centriole of various cells was measured and shown as scatter plots. Dots indicate individual samples, and horizontal lines and error bars indicate means and SDs, respectively. Total numbers of analyzed cells from a single experiment are shown (*n*). The immunostaining experiment was repeated three times to confirm the reproducibility. Statistical significances among multiple groups were calculated using one-way ANOVA followed by Tukey’s multiple comparison test. NS, not significant.

Recent studies using super-resolution microscopy and expansion microscopy demonstrated that the IFT-B and IFT-A complexes are localized at gaps between the DAPs of the basal body (Ishida *et al.*, 2021; Katoh *et al.*, 2020; Yang *et al.*, 2015, 2018), demonstrating that the localization of IFT-B and IFT-A is dependent on DAP proteins (Bowie *et al.*, 2018; Čajánek and Nigg, 2014; Kanie *et al.*, 2025; Mori *et al.*, 2025; Reed *et al.*, 2022; Schmidt *et al.*, 2012). On the other hand, analyses of cilia/flagella of various species using fluorescence recovery after photobleaching suggested that the IFT-A and IFT-B complexes are independently recruited to the basal body pool (Hibbard *et al.*, 2021; Mitra *et al.*, 2024; Wingfield *et al.*, 2017). Furthermore, we recently showed that the IFT-A, IFT-B, and dynein-2 complexes are independently assembled at the mother centriole/basal body before their incorporation into IFT trains (Tasaki *et al.*, 2025). In the course of this study, we noticed the possibility that assembly of the IFT-B complex and its recruitment to the mother centriole/basal body may be dependent on a specific subcomplex/subgroup of IFT-B and is independent of ciliogenesis (Tasaki *et al.*, 2025). Namely, although human telomerase reverse transcriptase-immortalized retinal pigment epithelial 1 (hTERT-RPE1) cells that are knocked out of *IFT81* (an IFT-B1a subunit) have impaired ciliogenesis, IFT88 (an IFT-B1b subunit) and IFT38 (an IFT-B2 subunit) were recruited to the mother centriole in these cells (see Fig. 1I and Fig. 2I). In the present study, we therefore extended our previous study and aimed to clarify the process of IFT-B complex assembly and recruitment to the mother centriole by investigating the localization of IFT-B2, IFT-B1b, and IFT-B1a subunits and their interactions under individual IFT-B subunit knockout conditions.

## Materials and Methods

### Plasmids, antibodies, chemicals, and KO cell lines

Plasmids for the preparation of lentiviral vectors for the expression of IFT-B subunits, and for production of the glutathione *S*-transferase (GST)-tagged anti-GFP nanobody (Nb) are listed in Table S1. The antibodies used in this study are also listed in Table S1. The KO cell lines used in this study are listed in Table S2; all of them were established from hTERT-RPE1 cells in our previous studies (Funabashi *et al.*, 2018; Hiyamizu *et al.*, 2023; Ishida *et al.*, 2022; Katoh *et al.*, 2017; Nozaki *et al.*, 2019; Tasaki *et al.*, 2023; Zhou *et al.*, 2022).

### Immunofluorescence analysis

Parental hTERT-RPE1 cells (American Type Culture Collection, CRL-4000) and KO cells were cultured in DMEM/Ham’s F-12 medium supplemented with 10% fetal bovine serum (FBS) and 0.348% sodium bicarbonate at 37°C in 5% CO_2_. To induce ciliogenesis under serum-starved conditions, the cells were grown on coverslips to 100% confluence, and cultured for 24 h in Opti-MEM (Thermo Fisher Scientific) containing 0.2% bovine serum albumin. For immunostaining, the cells were fixed and permeabilized with 3% paraformaldehyde for 5 min at 37°C, and further by methanol for 5 min at –20°C for IFT38, IFT88, and IFT81. The immunostained cells were observed using an Axio Observer microscope (Carl Zeiss).

To quantify the fluorescence intensities, all images in a set of experiments were acquired under the same setting and analyzed using the Zeiss ZEN 3.1 software. A region of interest (ROI) was constructed around the CEP43 signal using a drawing tool, and the fluorescence intensity in the ROI was quantified. To correct for local background intensity, the fluorescence intensity of a nearby region was subtracted. Statistical analyses were performed using GraphPad Prism version 10.1.2 software (GraphPad Software).

### Preparation of lentiviral vectors and establishment of stable cell lines

Lentiviral vectors for the stable expression of EGFP-fused IFT38, IFT52, and IFT74 were prepared as described previously (Takahashi *et al.*, 2012). Briefly, HEK293T cells (RBC2202, RIKEN BioResource Research Center) were cultured in DMEM with high glucose supplemented with 5% FBS. An EGFP-fused IFT-B construct in the pRRLsinPPT vector was transfected into HEK293T cells together with the packaging plasmids (pRSV-REV, pMD2.g, and pMDLg/pRRE; kind gifts from Peter McPherson, McGill University) (Thomas *et al.*, 2009) using Polyethylenimine Max (Polysciences). Eight hours after the transfection, the culture medium was replaced with fresh medium. The medium containing viral particles was then collected at 24, 36, and 48 h after transfection, passed through a 0.45-μm filter, and centrifuged at 32,000 × *g* at 4°C for 4 h. The precipitated lentiviral particles were resuspended in DMEM/Ham’s F-12 medium, and stored at –80°C until use. KO cells stably expressing an EGFP-fused construct were prepared by addition of the lentiviral suspension to the culture medium, followed by selection of the cells in culture medium containing blasticidin S (10 μg/mL; InvivoGen) or Zeocin (10 μg/mL; InvivoGen).

### Coimmunoprecipitation analysis

Cells stably expressing either EGFP-fused IFT38, IFT52, or IFT74 were grown to 100% confluence on a 6-cm plate, and cultured for 24 h in Opti-MEM containing 0.2% bovine serum albumin. These cells were then lysed in 300 μL of lysis buffer (20 mM HEPES–KOH [pH 7.4], 100 mM KCl, 5 mM NaCl, 3 mM MgCl_2_, 1 mM dithiothreitol, 10% glycerol and 0.1% Triton X-100) containing EDTA-free protease inhibitor cocktail (Nacalai Tesque) by placing on ice for 20 min. After centrifugation of the lysates at 16,100 × *g* at 4°C for 15 min, the supernatants were incubated with 5 μL of GST-tagged anti-GFP Nb prebound to glutathione-Sepharose 4B beads. After brief centrifugation in a microcentrifuge, the beads were washed three times with the lysis buffer and boiled in SDS-PAGE sample buffer. Proteins bound to the beads were separated by SDS-PAGE, and electroblotted onto an Immobilon-P membrane (Merck Millipore). The membrane was blocked in 5% skimmed milk and incubated sequentially with the primary antibody and the peroxidase-conjugated secondary antibody. Protein bands were detected with a Chemi Lumi one L kit or Chemi-Lumi super kit (Nacalai Tesque).

## Results

### Differential effects of the lack of the IFT-B1a, IFT-B1b, or IFT-B2 subunit on recruitment of the remaining sets of IFT-B subunits to the mother centriole

In the present study, we used hTERT-RPE1 cells knocked out of *IFT74* and *IFT81* (IFT-B1a subunits), of *IFT52* and *IFT88* (IFT-B1b subunits), and of *IFT38* and *IFT54* (IFT-B2 subunits) (Hiyamizu *et al.*, 2023; Ishida *et al.*, 2022; Katoh *et al.*, 2017; Nozaki *et al.*, 2019; Tasaki *et al.*, 2023; Zhou *et al.*, 2022) to investigate whether the absence of one of the IFT-B subunits affects recruitment of the other subunits to the mother centriole. In addition, we also used cells knocked out of *KIF3B*, a subunit of heterotrimeric kinesin II (Funabashi *et al.*, 2018), as a control of cells lacking cilia but with normal IFT-B complex assembly (Tasaki *et al.*, 2025).

We first investigated whether IFT38 (an IFT-B2 subunit) is able to localize to the mother centriole/basal body in these KO cells. In control RPE1 cells, IFT38 was primarily localized at the ciliary base, with a minor proportion within cilia (Fig. 1B). In *KIF3B*-KO cells, which lack cilia, IFT38 was localized at one of the two CEP43-positive centrioles (Fig. 1C), confirming that the IFT-B complex is recruited to the mother centriole independently of ciliogenesis (Tasaki *et al.*, 2025). In *IFT54*-KO cells as well as in *IFT38*-KO cells, IFT38 signals were undetectable at either of the centrioles (Fig. 1D, E; also see Fig. 1J), indicating that incorporation of IFT38 into the IFT-B complex and/or its recruitment to the mother centriole is dependent on IFT54, another IFT-B2 subunit (see Fig. 1A). In *IFT52*-KO cells and *IFT88*-KO cells, IFT38 was not detectable at the centrioles (Fig. 1F, G; also see Fig. 1J), indicating that mother centriole recruitment of IFT38 is dependent on the IFT-B1b subunits. By contrast, IFT38 signals were found at one of the two centrioles in *IFT74*-KO and *IFT81*-KO cells (Fig. 1H, I; also see Fig. 1J), although ciliogenesis was abrogated. Thus, recruitment of IFT38 to the mother centriole is independent of the IFT-B1a subunits, although the IFT74 and IFT81 subunits are essential for cilia formation owing to their role in tubulin transport (Bhogaraju *et al.*, 2013; Kubo *et al.*, 2016).

We then investigated the localization of IFT88 (an IFT-B1b subunit) in IFT-B KO cells. Like IFT38, IFT88 was mainly localized at the ciliary base, with a minor proportion within cilia in control RPE1 cells (Fig. 2B), and at one of the two CEP43-positive centrioles in *KIF3B*-KO cells (Fig. 2C). IFT88 signals were not detected at either of the centrioles in either *IFT52*-KO or *IFT88*-KO cells (Fig. 2F, G; also see Fig. 2J). In addition, IFT88 signals were not detected in the absence of IFT38 or IFT54 (Fig. 2D, E; also see Fig. 2J). By contrast, IFT88 was found localized at one of the two centrioles in *IFT74*-KO and *IFT81*-KO cells (Fig. 2H, I; also see Fig. 2J). Taken together with the data shown in Fig. 1, these observations indicate that the IFT-B2 subcomplex and the IFT-B1b subgroup are recruited to the mother centriole in a mutually dependent manner, but independently of the IFT-B1a subgroup.

**Fig. 3:**
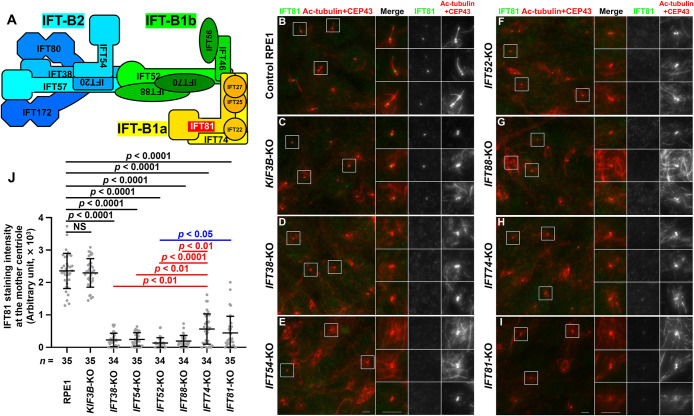
Components of IFT-B2 and IFT-B1b as well as IFT-B1a are required for mother centriole recruitment of IFT81 (A) Model diagram of assembly of the IFT-B complex with IFT81 highlighted in red. (B–I) Control RPE1 (B), *KIF3B*-KO (C), *IFT38*-KO (D), *IFT54*-KO (E), *IFT52*-KO (F), *IFT88*-KO (G), *IFT74*-KO (H), and *IFT81*-KO (I) cells were cultured under serum-starved conditions for 24 h to induce ciliogenesis, and immunostained with antibodies against IFT81 and Ac-tubulin + CEP43. Images shown on the right side are 2.5-times enlarged images of the boxed regions. Scale bars, 5 μm. (J) Staining intensity of IFT81 at the mother centriole of various cells was measured and shown as scatter plots. Dots indicate individual samples, and horizontal lines and error bars indicate means and SDs, respectively. Total numbers of analyzed cells from a single experiment are shown (*n*). The immunostaining experiment was repeated twice to confirm the reproducibility. Statistical significances among multiple groups were calculated using one-way ANOVA followed by Tukey’s multiple comparison test. NS, not significant.

**Fig. 4:**
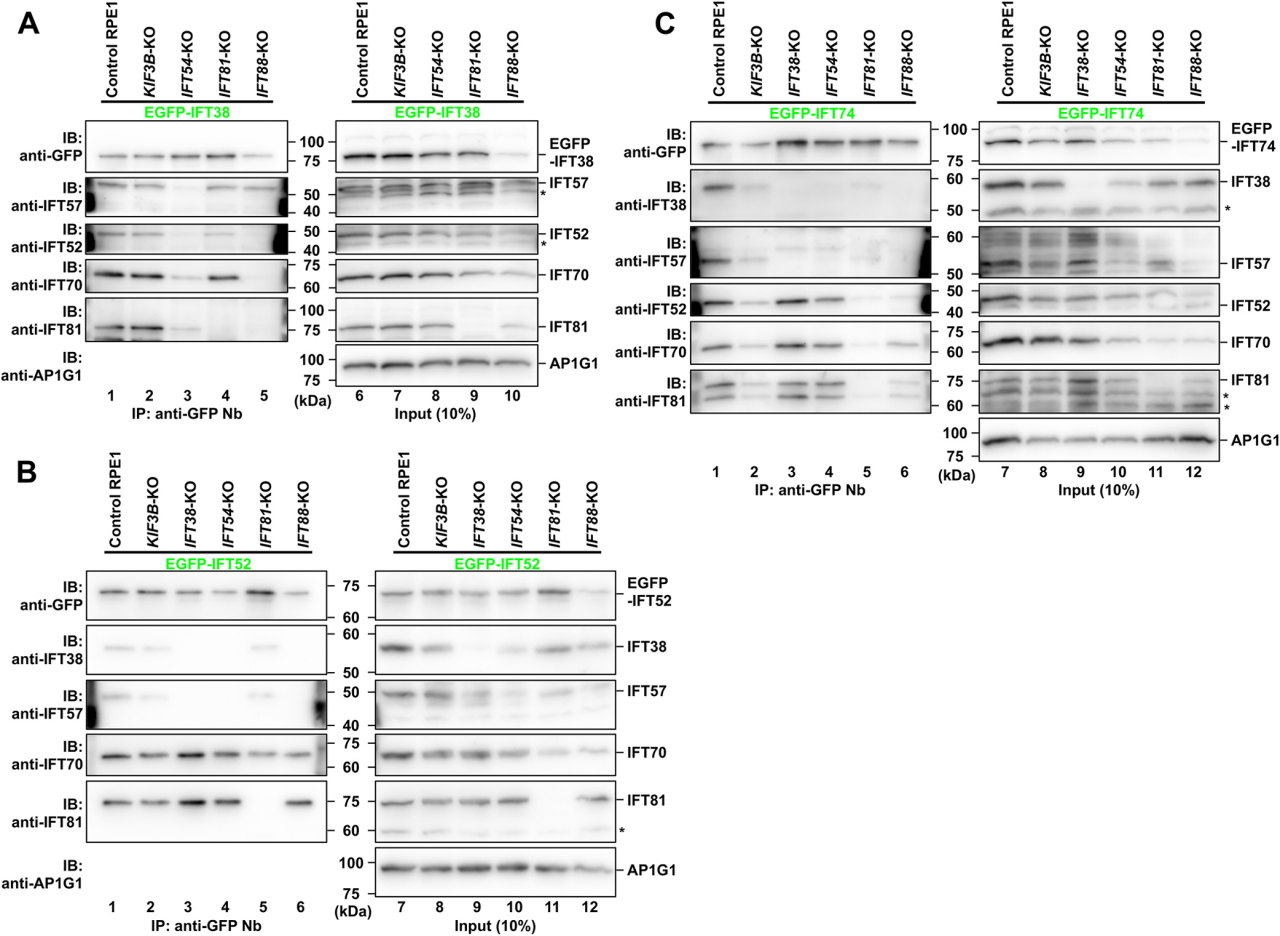
Differential effects of the lack of either the IFT-B1a, IFT-B1b, or IFT-B2 subunit on assembly of the remaining sets of IFT-B subunits EGFP-fused IFT38 (A), IFT52 (B), or IFT74 (C) was stably expressed in control RPE1 cells, and in *KIF3B*-KO, *IFT38*-KO, *IFT54*-KO, *IFT81*-KO, and *IFT88*-KO cells as indicated. Lysates were prepared from the cells and processed for immunoprecipitation with GST-tagged anti-GFP Nb, followed by SDS-PAGE and immunoblotting analysis using antibodies against GFP, IFT38, IFT57, IFT52, IFT70, and IFT81, as indicated. γ-Adaptin (AP1G1) was used as a loading control. Asterisks indicate the positions of nonspecific bands. Blots shown are representative of two experiments.

**Fig. 5:**
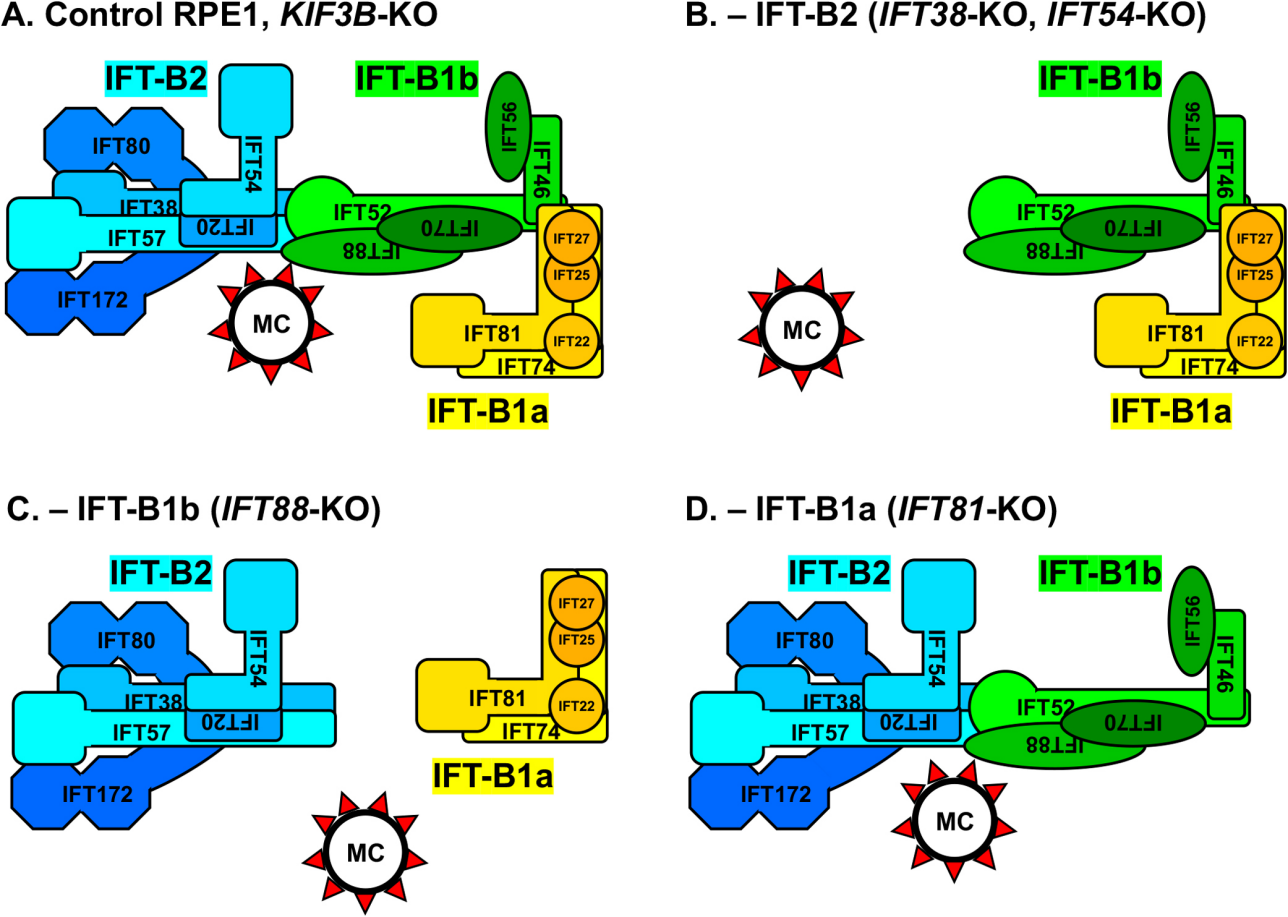
Model diagrams of the assembly and mother centriole recruitment of the IFT-B complex in cells lacking specific IFT-B subunits Model diagrams of the assembly and recruitment to the mother centriole of the IFT-B2 subcomplex, and IFT-B1b and IFT-B1a subgroups in control RPE1 cells (A), cells lacking an IFT-B2 subunit (B; IFT38 or IFT54), cells lacking an IFT-B1b subunit (C; IFT88), and cells lacking an IFT-B1a subunit (D; IFT81). MC, mother centriole. Red triangles indicate DAPs.

We also investigated the localization of IFT81 (an IFT-B1a subunit) in IFT-B KO cells. Like IFT38 and IFT88, IFT81 was mainly localized at the ciliary base with a minor proportion within cilia in control RPE1 cells, and at one of the two centrioles in *KIF3B*-KO cells (Fig. 3B, C). In *IFT38*-KO, *IFT54*-KO, *IFT52*-KO, and *IFT88*-KO cells, IFT81 signals were not detectable at the mother or daughter centriole (Fig. 3D–G; also see Fig. 3J), indicating that the mother centriole localization of IFT-B1a is dependent not only on IFT-B1b but also on IFT-B2, which is not directly linked to IFT-B1a (see Fig. 5A). However, it should be mentioned that, in *IFT74*-KO and *IFT81*-KO cells, IFT81 signals at the centriole were substantially decreased (Fig. 3H, I; also see Fig. 3J), but slight IFT81 staining remained (Fig. 3J). By immunoblotting analysis of lysates from *IFT81*-KO cells with anti-IFT81 antibody, we detected residual non-specific bands (see Fig. 4B, fifth panel, lane 11; and Fig. 4C, sixth panel, lane 11). Thus, although we do not know the exact reason, the residual IFT81 staining in *IFT74*-KO and *IFT81*-KO cells is probably inherent to the used IFT81 antibody.

### Differential effects of the lack of the IFT-B1a, IFT-B1b, or IFT-B2 subunit on assembly of the remaining sets of IFT-B subunits

Next, we investigated whether the lack of one of the IFT-B subunits affects assembly of the remaining sets of the IFT-B subunits. To this end, we expressed one of the IFT-B subunits fused to EGFP in cells knocked out of one of the IFT-B subunits. Then, to determine if the EGFP-fused subunit can be incorporated into the IFT-B complex, lysates prepared from the cells were processed for immunoprecipitation with GST-tagged anti-GFP Nb prebound to glutathione-Sepharose beads (Katoh *et al.*, 2015), followed by SDS-PAGE and immunoblotting analysis using antibodies against the various IFT-B subunits; however, it should be noted that the EGFP tag may interfere with the incorporation of the subunit into the complex.

To determine the assembly process of the IFT-B complex, we first expressed EGFP-fused IFT38 (an IFT-B2 subunit) in cells knocked out of one of the IFT-B subunits (Fig. 4A). When expressed in *KIF3B*-KO cells as the cilia-lacking control (Fig. 4A, lane 2), EGFP-IFT38 was able to coimmunoprecipitate IFT57 (an IFT-B2 subunit), IFT52 and IFT70 (IFT-B1b subunits), and IFT81 (an IFT-B1a subunit), indicating that EGFP-IFT38 was incorporated into the IFT-B holocomplex even in the absence of cilia formation. In cells knocked out of *IFT54* (an IFT-B2 subunit) (Fig. 4A, lane 3), not only the amount of IFT57, but also the amounts of IFT52, IFT70, and IFT81 that were coimmunoprecipitated with EGFP-IFT38 were substantially reduced, suggesting that the IFT-B2 subcomplex was not normally formed in the absence of IFT54, and thereby EGFP-IFT38 was unable to coprecipitate the IFT-B1b and IFT-B1a subunits. In cells knocked out of *IFT88* (an IFT-B1b subunit) (Fig. 4A, lane 5), EGFP-IFT38 was able to coprecipitate IFT57, indicating that the IFT-B2 subcomplex was normally formed even in the absence of the IFT-B1b subgroup. By contrast, EGFP-IFT38 was unable to coprecipitate IFT81 as well as IFT52 and IFT70, indicating that the IFT-B2 subcomplex and the IFT-B1a subgroup were not included in the same complex if the IFT-B1b subgroup was not present. In cells knocked out of *IFT81* (an IFT-B1a subunit) (Fig. 4A, lane 4), EGFP-IFT38 was able to coprecipitate IFT57, IFT52, and IFT70, indicating that the IFT-B2 subcomplex and the IFT-B1b subgroup were linked to each other in the absence of the IFT-B1a subgroup. This is consistent with our previous studies showing that IFT38 and IFT57 from IFT-B2, and IFT52 and IFT88 from IFT-B1b are minimally required for the IFT-B2–IFT-B1b interaction (Katoh *et al.*, 2016; Kobayashi *et al.*, 2021).

We then analyzed the IFT-B KO cells expressing EGFP-fused IFT52 (an IFT-B1b subunit) (Fig. 4B). In *KIF3B*-KO cells (lane 2) as well as in control RPE1 cells (lane 1), all the analyzed IFT-B subunits were coprecipitated with EGFP-IFT52, indicating that EGFP-IFT52 is incorporated into the IFT-B holocomplex independently of cilia formation. In cells knocked out of *IFT38* (lane 3) or *IFT54* (lane 4) (IFT-B2 subunits), EGFP-IFT52 coprecipitated IFT70 (an IFT-B1b subunit) and IFT81 (an IFT-B1a subunit), indicating that the IFT-B1b subgroup is linked to the IFT-B1a subgroup even in the absence of the IFT-B2 subcomplex. In *IFT81*-KO cells (lane 5), EGFP-IFT52 was able to coprecipitate not only IFT70 but also IFT38 and IFT57, indicating that the IFT-B1b subgroup is linked to the IFT-B2 subcomplex in the absence of the IFT-B1a subgroup. In *IFT88*-KO cells (lane 6), neither IFT38 nor IFT57 was coprecipitated with EGFP-IFT52, in agreement with previous studies showing that the IFT52–IFT88 dimer of the IFT-B1b subgroup constitutes the interface with the IFT-B2 subcomplex (Katoh *et al.*, 2016; Kobayashi *et al.*, 2021; Petriman *et al.*, 2022; Taschner *et al.*, 2016).

We finally analyzed IFT-B KO cells expressing EGFP-fused IFT74 (an IFT-B1a subunit) (Fig. 4C). Again, all the analyzed IFT-B subunits were coprecipitated with EGFP-IFT74 in *KIF3B*-KO cells (lane 2) as well as in control RPE1 cells (lane 1), indicating that EGFP-IFT74 was incorporated into the IFT-B holocomplex in a ciliogenesis-independent manner. In cells knocked out of *IFT38* (lane 3) or *IFT54* (lane 4) (IFT-B2 subunits), IFT52 and IFT70 (IFT-B1b subunits) as well as IFT81 (an IFT-B1a subunit) were coprecipitated with EGFP-IFT74, confirming that the IFT-B1a and IFT-B1b subgroups are linked to each other even in the absence of the IFT-B2 subcomplex. By contrast, in *IFT81*-KO cells, the coimmunoprecipitation with EGFP-IFT74 of all the IFT-B subunits analyzed was virtually undetectable (lane 5). These results using *IFT81*-KO cells expressing EGFP-IFT74 are in good agreement with the fact that IFT74 forms a tight heterodimer with IFT81 via their long coiled-coil regions, and interacts with the IFT46–IFT52 dimer of the IFT-B1b subgroup (Petriman *et al.*, 2022; Taschner *et al.*, 2014; Zhou *et al.*, 2022). In *IFT88*-KO cells (lane 6), IFT70 was coprecipitated with EGFP-IFT74. These results are consistent with the fact that the IFT74–IFT81 dimer interacts with the IFT46–IFT52 dimer of the IFT-B1b subgroup, to which IFT70 binds (Katoh *et al.*, 2016; Petriman *et al.*, 2022; Taschner *et al.*, 2014). IFT38 or IFT57 was not coprecipitated with EGFP-IFT74 in *IFT88*-KO cells, consistent with the fact that the IFT52–IFT88 dimer interacts with IFT38 and IFT57 of the IFT-B2 subcomplex (Katoh *et al.*, 2016; Kobayashi *et al.*, 2021; Petriman *et al.*, 2022; Taschner *et al.*, 2016).

## Discussion

We previously showed that the IFT-B, IFT-A, and dynein-2 complexes are independently recruited to the mother centriole (Tasaki *et al.*, 2025). In the present study, we set out to pursue the association between the assembly and localization to the mother centriole of the IFT-B complex, which can be divided into the IFT-B2 subcomplex and IFT-B1 subcomplex (IFT-B1a and IFT-B1b subgroups) (see Fig. 5A). Table 1 summarizes the results obtained in the present study, and Fig. 5 shows model diagrams of the assembly and mother centriole recruitment of the IFT-B complex in control RPE1 cells and in KO cells of each IFT subunit.

Immunofluorescence analysis of cells knocked out of individual IFT-B subunits (Fig. 1–3) and coimmunoprecipitation analysis of these KO cells expressing one of the IFT-B subunits fused to EGFP (Fig. 4) showed that, in the absence of an IFT-B1b subgroup subunit (IFT52 or IFT88), none of the analyzed IFT-B subunits could localize to the mother centriole, although the IFT-B2 subcomplex and the IFT-B1a subgroup appeared to be able to be assembled independently (Fig. 5C). Thus, given the central role of the IFT-B1b subgroup in bridging IFT-B2 and IFT-B1a (see Fig. 5A) (Petriman *et al.*, 2022) and the potential role of a tetramer unit involving IFT52 and IFT88 from IFT-B1b and IFT38 and IFT57 from IFT-B2 in the localization of IFT-B to the mother centriole (see below), cilia do not form in the absence of IFT-B1b owing to the lack of assembly of the IFT-B holocomplex.

In the absence of the IFT-B2 subunit (IFT38 or IFT54), the IFT-B1b and IFT-B1a subgroups can be linked to each other (Fig. 4B, C; also see Fig. 5B); namely, the IFT-B1 subcomplex is formed. This is in line with early biochemical studies of *Chlamydomonas* IFT-B proteins showing that the peripheral (IFT-B2) subunits associate with the core (IFT-B1) of the IFT-B complex (the IFT-B1 subcomplex) (Lucker *et al.*, 2005, 2010; Taschner *et al.*, 2011), and with previous coimmunoprecipitation studies of EGFP-fused and mCherry-fused IFT-B proteins showing that the IFT46–IFT52 dimer from IFT-B1b and IFT81 from IFT-B1a are involved in the IFT-B1b–IFT-B1a linkage (Katoh *et al.*, 2016; Tasaki *et al.*, 2023; Zhou *et al.*, 2022). However, neither the IFT-B1b (IFT88) subunit nor the IFT-B1a (IFT81) subunit can localize to the mother centriole, indicating that the IFT-B1b–IFT-B1a linkage (in other words, the IFT-B1 subcomplex) is not sufficient for localization of the IFT-B complex to the mother centriole (Fig. 5B). In the absence of the IFT-B2 subcomplex, the IFT-B complex cannot be recruited to the mother centriole, resulting in the absence of ciliogenesis.

By contrast, in the absence of the IFT-B1a subunit (IFT74 or IFT81), the IFT-B2 subcomplex and IFT-B1b subgroup can be linked to each other (Fig. 5D), which is consistent with previous studies showing that the tetramer unit composed of IFT38 and IFT57 from IFT-B2, and IFT52 and IFT88 from IFT-B1b is crucial for the linking of the IFT-B2 and IFT-B1 subcomplexes (Katoh *et al.*, 2016; Kobayashi *et al.*, 2021; Taschner *et al.*, 2016). In addition, both IFT-B2 (IFT38) and IFT-B1b (IFT88) can localize to the mother centriole in the absence of IFT-B1a (Fig. 5D). Thus, it is likely that the IFT-B2–IFT-B1b linkage is essential for the mother centriole localization of the IFT-B complex; this was somewhat unexpected because early biochemical studies on *Chlamydomonas* IFT-B proteins indicated that the IFT-B peripheral (IFT-B2) proteins are associated with the core (IFT-B1) of the IFT-B complex (Lucker *et al.*, 2005, 2010; Taschner *et al.*, 2011). However, even with the recruitment of IFT-B2–IFT-B1b to the mother centriole, cilia do not form in the absence of IFT74 or IFT81. This is probably owing to the crucial role of the IFT74–IFT81 dimer in the transport of tubulin dimers for extension and maintenance of axonemal microtubules (Bhogaraju *et al.*, 2013; Kubo *et al.*, 2016).

Thus, although ciliogenesis is inhibited in the absence of the analyzed individual IFT-B subunits, the factors involved in the inhibition are likely to vary depending on the missing subunits, including impaired recruitment of the IFT-B complex to the mother centriole and impaired tubulin transport. A key remaining question is which subunits participate in the localization of IFT-B to the mother centriole. One possibility is the tetramer unit involved in the linkage between IFT-B2 and IFT-B1b (IFT38 and IFT57 from IFT-B2, and IFT52 and IFT88 from IFT-B1b), as IFT-B2 or IFT-B1 (IFT-B1b + IFT-B1a) alone is not sufficient for the mother centriole localization of the IFT-B complex. As the tetramer unit constitutes the binding site of heterotrimeric kinesin-II (Funabashi *et al.*, 2018), one possible scenario is that the IFT-B complex is recruited to the mother centriole via the tetramer, and after leaving the mother centriole, kinesin-II occupies the tetramer and drives anterograde transport of the IFT trains.

Another question is what in the mother centriole determines the recruitment of the IFT-B complex. Previous studies showed that DAP proteins, including CEP164 and Tau-tubulin kinase 2 (TTBK2), are essential for recruitment of the IFT-A, IFT-B, and dynein-2 complexes to the mother centriole (Bowie *et al.*, 2018; Čajánek and Nigg, 2014; Goetz *et al.*, 2012; Kanie *et al.*, 2025; Mori *et al.*, 2025; Reed *et al.*, 2022; Schmidt *et al.*, 2012). A recent proximity labeling study on TTBK2 identified many ciliary, centrosomal, and pericentriolar material, as well as centriolar satellite proteins, suggesting direct or indirect interactions of these proteins with TTBK2; IFT46, IFT52, IFT74, and IFT81 were included in the TTBK2 proximity list (Nguyen and Goetz, 2023). However, our attempts to show direct interactions of TTBK2 with these IFT-B proteins have been unsuccessful to date (Mori *et al.*, 2025). On the other hand, we showed that the kinase activity and CEP164-dependent recruitment of TTBK2 are required for the localization of the IFT-A, IFT-B, and dynein-2 complexes to the mother centriole (Mori *et al.*, 2025). Recently, Kanie *et al.* reported that KO of *TTBK2* in RPE1 cells impaired the localization of several DAP proteins, including CEP83, FBF1, ANKRD26, PIDD1, and NCS1 (Kanie *et al.*, 2025). These proteins, which are phosphorylated by TTBK2, may hence be involved in the recruitment of the IFT-B complex to the mother centriole.

Thus, future issues to be addressed include which subunit(s) of the IFT-B complex determine its mother centriole localization and which protein(s) of the mother centriole interact with the IFT-B complex. The analyses we have employed to date, based on protein–protein interactions and the expression of various proteins in KO cells, will help to address these issues.

## Data availability

All data in this study are included in the article or the supplementary material.

## Author contributions

K.T. designed and performed the experiments and prepared the manuscript, and Y.K., H.-W.S., and K.N. designed the experiments and prepared the manuscript.

## Conflict of Interest

The authors declare that they have no competing interests associated with this study.

## Figures and Tables

**Table 1:** Summary of the results of the present study

Subcomplex/subgroup	Cell	Cilia formation	Basal body/mother centriole localization			Assembly	
			IFT-B2 (IFT38)	IFT-B1b (IFT88)	IFT-B1a (IFT81)	IFT-B2–IFT-B1b	IFT-B1b–IFT-B1a
	Control RPE1	+	+	+	+	+	+
	*KIF3B*-KO	–	+	+	+	+	+
IFT-B2	*IFT38*-KO	–	–	–	–	–	+
	*IFT54*-KO	–	–	–	–	–	+
IFT-B1b	*IFT52*-KO	–	–	–	–	NI	NI
	*IFT88*-KO	–	–	–	–	–	±*^1^
IFT-B1a	*IFT74*-KO	–	+	+	–	NI	NI
	*IFT81*-KO	–	+	+	–	+	–

NI: not investigated. *^1^ In *IFT88*-KO cells, there was partial interaction between IFT-B1a and the remaining IFT-B1b components (see Fig. 4C). We previously demonstrated that the IFT74–IFT81 dimer of the IFT-B1a subgroup can interact with the IFT46–IFT52 dimer of the IFT-B1b subgroup in the absence of IFT88 (Katoh *et al.*, 2016; Zhou *et al.*, 2022).

## Abbreviations

BBS: Bardet-Biedl syndrome
DAP: distal appendage
FBS: fetal bovine serum
GST: glutathione *S*-transferase
Hh: Hedgehog
hTERT-RPE1: human telomerase reverse transcriptase-immortalized retinal pigment epithelial 1
IFT: intraflagellar transport
KO: knockout
Nb: nanobody
ROI: region of interest
SMO: Smoothened
TTBK2: Tau-tubulin kinase 2
TZ: transition zone
