# Supplementary material for: Assembly and mother centriole recruitment of IFT-B subcomplexes to form IFT-B holocomplex
Source: Cell Struct Funct. 2025 Jun 24;50(2):157–68. doi: 10.1247/csf.25027 (PMC12967509; doi:10.1247/csf.25027)
Supplement: Supplementary file 1 — Supplementary Materials [file csf_50_25027_1.pdf]

Table S1. Plasmids, antibodies, and chemicals used in this study

Plasmid	Reference	Identifier
pRRLsinPPT-EGFP-C-hIFT38-IRES-Blast	This study	N/A
pRRLsinPPT-EGFP-C-hIFT52-IRES-Zeo	(Tasaki *et al.*, 2025)	N/A
pRRLsinPPT-EGFP-C-hIFT74-IRES-Blast	(Zhou *et al.*, 2022)	N/A
pRSV-REV	(Thomas *et al.*, 2009)	Gift from Peter McPherson
pMD2.g	(Thomas *et al.*, 2009)	Gift from Peter McPherson
pMDLg/pRRE	(Thomas *et al.*, 2009)	Gift from Peter McPherson
pGEX6P1-GFP-Nanobody	(Katoh *et al.*, 2015)	Addgene 61838
**Antibody (dilution and use)**	**Source or reference**	**Identifier**
Monoclonal mouse anti-Ac-α-tubulin (1:1,000 for IF)	Sigma-Aldrich	Clone 6-11B-1
Monoclonal mouse anti-CEP43 (1:10,000 for IF)	Abnova	Clone 2B1
Polyclonal rabbit anti-IFT38 (1:3,000 for IF; 1:5,000 for IB)	(Botilde *et al.*, 2013)	Gift from Hiroshi Hamada and Takanobu A. Katoh
Polyclonal rabbit anti-IFT57 (1:1,000 for IB)	Proteintech	11083-1-AP
Polyclonal rabbit anti-IFT70 (1:1,000 for IB)	Proteintech	25352-1-AP
Polyclonal rabbit anti-IFT81 (1:100 for IF)	BiCell Scientific	90606
Polyclonal rabbit anti-IFT81 (1:1,000 for IB)	Proteintech	11744-1-AP
Polyclonal rabbit anti-IFT88 (1:500 for IF)	Proteintech	13967-1-AP
Monoclonal mouse anti-GFP (1:10,000 for IB)	Proteintech	66002-1-Ig
Monoclonal mouse anti-γ-adaptin (1:500 for IB)	Sigma-Aldrich	Clone 100/3
AlexaFluor-conjugated secondary (1:1,000 for IF)	Molecular Probes	A11034, A21137, A21147
Peroxidase-conjugated secondary (1:5,000 for IB)	Jackson ImmunoResearch	115-035-166, 111-035-144

IF, immunofluorescence; IB, immunoblotting: N/A, not applicable

Table S2. KO cell lines used in this study

Knocked-out gene	Cell line	Reference
IFT38	#38-1-17	(Nozaki *et al.*, 2019)
IFT52	#52-2-8	(Ishida *et al.*, 2022)
IFT54	#54-4-3	(Hiyamizu *et al.*, 2023)
IFT74	#74-2-11	(Zhou et al., 2022)
IFT81	#81-1-4	(Tasaki *et al.*, 2023)
IFT88	#88-1-7	(Katoh *et al.*, 2017)
KIF3B	#3B-2-1	(Funabashi *et al.*, 2018)

Supplemental references

Funabashi, T., Katoh, Y., Okazaki, M., Sugawa, M., and Nakayama, K. 2018. Interaction of heterotrimeric kinesin-II with IFT-B-connecting tetramer is crucial for ciliogenesis. *J. Cell Biol.*, **217**: 2867-2876.

Hiyamizu, S., Qiu, H., Tsurumi, Y., Hamada, Y., Katoh, Y., and Nakayama, K. 2023. Dynein-2-driven intraciliary retrograde trafficking indirectly requires multiple interactions of IFT54 in the IFT-B complex with the dynein-2 complex. *Biol. Open*, **12**.

Ishida, Y., Tasaki, K., Katoh, Y., and Nakayama, K. 2022. Molecular basis underlying the ciliary defects caused by IFT52 variations found in skeletal ciliopathies. *Mol. Biol. Cell*, **33**: ar83.

Katoh, Y., Michisaka, S., Nozaki, S., Funabashi, T., Hirano, T., Takei, R., and Nakayama, K. 2017. Practical method for targeted disruption of cilia-related genes by using CRISPR/Cas9-mediated, homology-independent knock-in system. *Mol. Biol. Cell*, **28**: 898-906.

Katoh, Y., Nozaki, S., Hartanto, D., Miyano, R., and Nakayama, K. 2015. Architectures of multisubunit complexes revealed by a visible immunoprecipitation assay using fluorescent fusion proteins. *J. Cell Sci.*, **128**: 2351-2362.

Nozaki, S., Castro Araya, R.F., Katoh, Y., and Nakayama, K. 2019. Requirement of IFT-B–BBSome complex interaction in export of GPR161 from cilia. *Biol. Open*, **8**: bio043786.

Tasaki, K., Satoda, Y., Chiba, S., Shin, H.W., Katoh, Y., and Nakayama, K. 2025. Mutually independent and cilia-independent assembly of IFT-A and IFT-B complexes at mother centriole. *Mol. Biol. Cell*, **36**: ar48.

Tasaki, K., Zhou, Z., Ishida, Y., Katoh, Y., and Nakayama, K. 2023. Compound heterozygous IFT81 variations in a skeletal ciliopathy patient cause Bardet-Biedl syndrome-like ciliary defects. *Hum. Mol. Genet.*, **32**: 2887-2900.

Thomas, S., Ritter, B., Verbich, D., Sanson, C., Bourbonnière, L., McKinney, R.A., and McPherson, P.S. 2009. Intersectin regulates dendritic spine development and somatodendritic endocytosis but not synaptic vesicle recycling in hippocampal neurons. *J. Biol. Chem.*, **284**: 12410-12419.

Zhou, Z., Qiu, H., Castro-Araya, R.-F., Takei, R., Nakayama, K., and Katoh, Y. 2022. Impaired cooperation between IFT74/BBS22–IFT81 and IFT25–IFT27/BBS19 in the IFT-B complex causes ciliary defects in Bardet-Biedl syndrome. *Hum. Mol. Genet.*, **31**: 1681-1693.
